# Alumina Extraction from Coal Fly Ash via Pre-Desilication, Vacuum Reduction, and the Alkali Dissolving Method

**DOI:** 10.3390/ma19132909

**Published:** 2026-07-07

**Authors:** Teng Li, Yao Chen, Xing Chen, Haitao Yuan, Tao Xiong, Wenzhou Yu

**Affiliations:** College of Materials Science and Engineering, Chongqing University, Chongqing 400045, China; 202509131349@stu.cqu.edu.cn (T.L.); 202409021150t@stu.cqu.edu.cn (Y.C.); 20212983@stu.cqu.edu.cn (X.C.); m15877867328@163.com (H.Y.); 20230901001z@stu.cqu.edu.cn (T.X.)

**Keywords:** coal fly ash, pre-desilication, vacuum reduction, alkali dissolving, alumina

## Abstract

**Highlights:**

- A new strategy for alumina extraction from CFA by integrating pre-desilication was proposed.
- Pre-desilication process increased Al_2_O_3_/SiO_2_ ratio of CFA from 0.78 to 1.27.
- Over 90% of Al_2_O_3_ was extracted by alkali dissolving.
- Material and energy consumption was reduced by 30.25% and 35.18%, respectively, in this process.

**Abstract:**

The high silica content of coal fly ash (CFA) poses a significant challenge for alumina extraction, resulting in high material and energy consumption. To reduce the silica content and improve alumina extraction efficiency, a novel process combining pre-desilication, vacuum reduction, and alkali dissolving is proposed. In the pre-desilication stage, amorphous silica in CFA is effectively removed by NaOH solution, increasing the Al_2_O_3_/SiO_2_ mass ratio from 0.78 to 1.27. The desilicated coal fly ash (D-CFA) is then subjected to vacuum carbothermal reduction with the addition of Fe_2_O_3_ and CaO to produce Fe-Si alloys and CaO·xAl_2_O_3_. The resulting CaO·xAl_2_O_3_ can be dissolved via alkali dissolving to extract alumina, achieving an alumina dissolving rate of over 90%. The Fe-Si alloys remaining in the dissolved residue are subsequently recovered by magnetic separation. Compared with the process without pre-desilication, the current process reduces material input by 30.25% and energy consumption by 35.18%, demonstrating that this approach offers a low-cost, energy-efficient, and environmentally friendly route for high-value-added utilization of CFA.

## 1. Introduction

With the rapid development of the electric power industry, reliance on conventional fossil energy, especially coal, has intensified year by year, even though the exploration of renewable energy has been greatly strengthened. According to statistics [1], coal-fired power accounted for 67.36% of the total electricity generation in China at the year of 2024, and this upward trend is expected to continue for several years, owing to the development of electric vehicles and other electric driving equipment. Consequently, coal-fired by-products, particularly coal fly ash (CFA), are discharged on a massive scale (over 800 million tons in 2024 in China) [2]. Although some encouragement policies have promoted the comprehensive utilization of CFA, a significant portion remains untreated, making CFA a major solid waste pollutant. From a long-term perspective, the stockpiling of CFA will pose threats to both the ecological environment and public health [3]. For example, fine CFA particles may result in atmospheric pollution due to their wind-dispersed nature. Additionally, leachable heavy metals in CFA, such as Pb, Cd, and Hg [4,5], can contaminate water bodies and cause soil degradation. Therefore, achieving efficient and high-value utilization of CFA is of paramount importance for environmental protection and sustainable resource management [6].

Conventionally, CFA is mainly used in construction materials, such as cement and concrete. However, the low-value-added nature of these products limits their ability to expand the applications of CFA. From a compositional perspective, CFA is notably rich in aluminum (Al), silicon (Si), lithium (Li), gallium (Ga) and rare earth elements (REEs). In some regions of China, vast reserves of high-alumina CFA are stockpiled near thermal power plants [7], with Al_2_O_3_ content ranging from 40 to 55 wt.% [8,9]. This type of CFA is equivalent to medium-grade bauxite, and its prospective reserves could be ten billion tons according to some reports [8,10,11]. Therefore, extracting alumina from CFA would not only achieve waste valorization but also alleviate dependency on conventional bauxite resources.

The established technologies for extracting alumina from CFA can be broadly divided into alkaline sintering and acidic leaching methods. The alkaline sintering process, through, for example, lime-sintering or soda-lime sintering methods, is relatively mature. It decomposes stable phases like mullite through high-temperature sintering with additives such as CaCO_3_ and Na_2_CO_3_. However, this process requires excessive amounts of lime to fix silica, leading to the generation of large quantities of calcium silicate slag and high energy consumption [12]. In contrast, acid leaching methods operate at lower temperatures but face challenges such as co-leaching of impurities (e.g., Fe and Ti) and severe equipment corrosion [13]. Up to now, neither alkaline nor acidic methods have realized large-scale industrial application. A common challenge for these routes is that, particularly for CFA derived from pulverized coal furnaces, the aluminum in CFA is predominantly locked in the chemically inert crystalline phase of mullite (3Al_2_O_3_·2SiO_2_) [14]. Given the stability of Al-O-Si bonds, decomposing the mullite phase requires harsh conditions, resulting in high energy input, excessive consumption of chemical reagents, and the generation of substantial secondary waste.

In our prior research [15,16,17,18,19], a vacuum carbothermal reduction method employing Fe_2_O_3_ and CaO as the additives was proposed. This method converts silica into Fe-Si alloys and transforms alumina into a calcium aluminate phase, thereby enabling the extraction of alumina in the subsequent steps, such as alkali dissolving. This strategy effectively achieves the separation of Al and Si and the decomposition of the stable mullite phase in CFA. Moreover, the vacuum environment successfully lowers the reduction temperature and reduces energy consumption. However, a critical limitation remains in this process: the high silica content in raw CFA necessitates a large amount of Fe_2_O_3_ additive to form a separable Fe-Si alloys phase [18], which significantly reduces the alumina yield per batch of furnace charge. Consequently, higher material costs and lower energy efficiency will emerge in this process if the silica content is high. Therefore, the silica content should be reduced to make this process more economical and efficient. To address this issue, pre-desilication of CFA would be a possible solution. In the previous study, the pre-desilication technology has been extensively employed to decrease the silica content in CFA by selectively removing amorphous SiO_2_ using alkaline solution. Bai [20] et al. showed that after pre-desilication, the Al_2_O_3_/SiO_2_ mass ratio of CFA was elevated from 0.86 to 1.63, which substantially reduced the amount of additives required in the subsequent lime–soda sintering process. Liu [21] et al. reported that under optimized conditions (95 °C, 15 wt.% NaOH, reaction time of 1 h), the desilication efficiency reached 48.6%, increasing the Al_2_O_3_/SiO_2_ ratio from 1.27 to 2.23. In another study, Xing [22] et al. developed an alkali pre-desilication enhanced mechanochemical extraction process for high alumina fly ash (HAFA), successfully increasing the Al/Si mass ratio of the valuable aluminum-rich residue to 2.51, significantly outperforming the desilication efficiency of conventional methods. Moreover, the desilication liquor (rich in sodium silicate) can be carbonated to produce precipitated silica (white carbon black). Li [23] et al. conducted a systematic investigation on the preparation of white carbon black from the desilicated solution of high alumina fly ash via the carbonation method. The obtained product (BTH 01) satisfied the type A standard of white carbon black, achieving high value utilization of the silicon component while enabling the closed-loop recycling of the alkaline solution, thereby significantly reducing reagent consumption and waste discharge.

The above studies collectively confirm that pre-desilication is an effective and feasible method to reduce downstream reagent consumption and energy demand. Hence, it is believed that if the pre-desilication can be employed before the vacuum thermal reduction, it will make the alumina extraction process more practical. However, several new challenges remain in this process. For instance, the mineralogical phase of the CFA will change after the pre-desilication, which will introduce difficulty and uncertainty for the next step, e.g., vacuum thermal reduction. Additionally, linking this process efficiently would also represent a challenge for its large-scale application. All these issues are expected to be resolved by relying on comprehensive research. Based on the analysis above, a new strategy for extracting alumina from CFA employing pre-desilication, vacuum carbothermal reduction, and alkali dissolving is proposed in this work. To demonstrate the feasibility of this integrated process, fundamental investigations were systematically conducted, including the dissolving mechanism of amorphous SiO_2_ during pre-desilication, the thermodynamic behavior and phase transformation characteristics of desilicated CFA during vacuum reduction, and the separation mechanisms of valuable elements in the subsequent alkali-dissolving process. Finally, a comprehensive comparison of energy consumption and material consumption with and without pre-desilication was carried out.

## 2. Materials and Methods

### 2.1. Raw Materials

**Figure 1:**
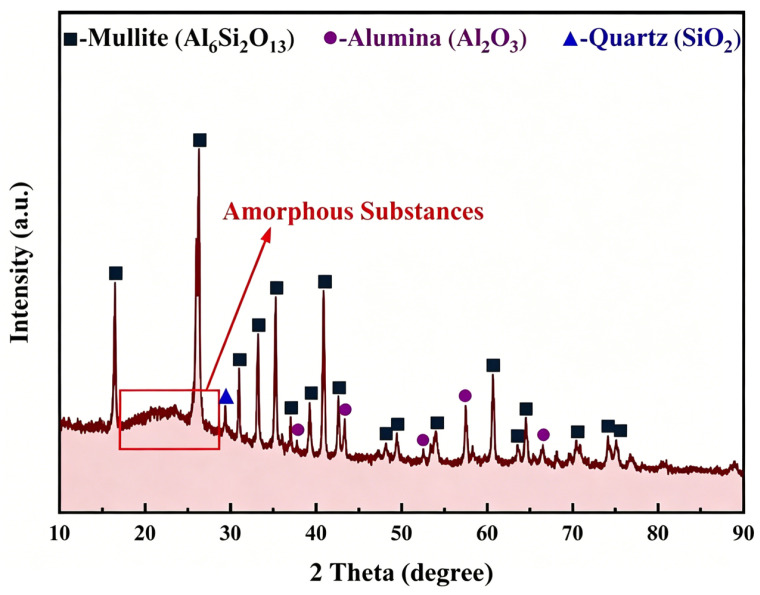
X-ray diffraction analysis of coal fly ash.

The CFA used in this study was collected from a thermal power plant in Inner Mongolia, China. Its mineral phases, determined by X-ray diffraction (XRD, Rigaku D/max 2500 PC; Rigaku Corporation, Tokyo, Japan), were mainly mullite (Al_6_Si_2_O_13_), corundum (Al_2_O_3_), and quartz (SiO_2_), together with an amorphous hump characteristic of glassy phases (Figure 1). The chemical composition, analyzed by X-ray fluorescence (XRF, Shimadzu XRF-1800; Shimadzu Corporation, Kyoto, Japan), is listed in Table 1. The Al_2_O_3_ and SiO_2_ contents were 38.97 wt.% and 49.89 wt.%, respectively, giving an initial Al_2_O_3_/SiO_2_ mass ratio of 0.78. Bituminous coal with a fixed carbon content of 71.22% was used as the reducing agent (Table 2). All chemical reagents (Na_2_CO_3_, Fe_2_O_3_, CaO, NaOH) were of analytical grade and purchased from Aladdin. The PDF/JCPDS card numbers used for phase identification of all samples (raw CFA, D-CFA, reduced products, and dissolved residues) in this study are summarized in Table 3.

### 2.2. Pre-Desilication of Coal Fly Ash

Pre-desilication was performed to selectively remove amorphous silica. In a typical run, 5 g of CFA was mixed with 100 mL of NaOH solution (concentration ranging from 25 to 150 g/L) in a 250 mL polytetrafluoroethylene (PTFE) beaker. The suspension was stirred at 300 rpm and heated at a desired temperature (30–90 °C) for 2 h using a thermostatically controlled heating magnetic stirrer. Experiments at 110 °C were carried out in a miniature high-pressure reactor. After reaction, the solid residue was filtered, washed three times with deionized water, and dried at 100 °C to constant weight. The dissolution efficiencies of SiO_2_ and Al_2_O_3_ were calculated from the mass change and XRF analysis of the residue. The optimum conditions (100 g/L NaOH, 90 °C, L/S ratio 20, 2 h) were selected for preparing desilicated CFA for subsequent reduction.

### 2.3. Vacuum Carbothermic Reduction

The desilicated coal fly ash (D-CFA) was mixed with bituminous coal, Fe_2_O_3_, Na_2_CO_3_ and CaO according to the designed ratios. The mixture was homogenized in a ball mill at 300 rpm for 10 min and then pressed into pellets (20 mm diameter, 20 MPa, 1 min). The pellets were dried at 393 K for 12 h. Reduction was performed in a vacuum furnace (pressure: ~100 Pa) at a heating rate of 10 K/min. After reaching the target temperature (1423–1573 K) the samples were held for 2–8 h and then cooled under vacuum. The effects of CaO/Al_2_O_3_ molar ratio and Na_2_CO_3_ addition on the mineralogical evolution were systematically investigated.

### 2.4. Recovery of Fe-Si Alloys and Al_2_O_3_

The reduced product was ground and subjected to alkali dissolving in a mixed solution of Na_2_CO_3_ and NaOH. After leaching, the slurry was filtered. The leachate was collected for analysis, and the solid residue was dried. The alumina dissolving efficiency was calculated from the Al_2_O_3_ concentration in the leachate, as determined by inductively coupled plasma–optical emission spectrometry (ICP-OES) and the Al_2_O_3_ content in the reduced sample.

**Figure 2:**
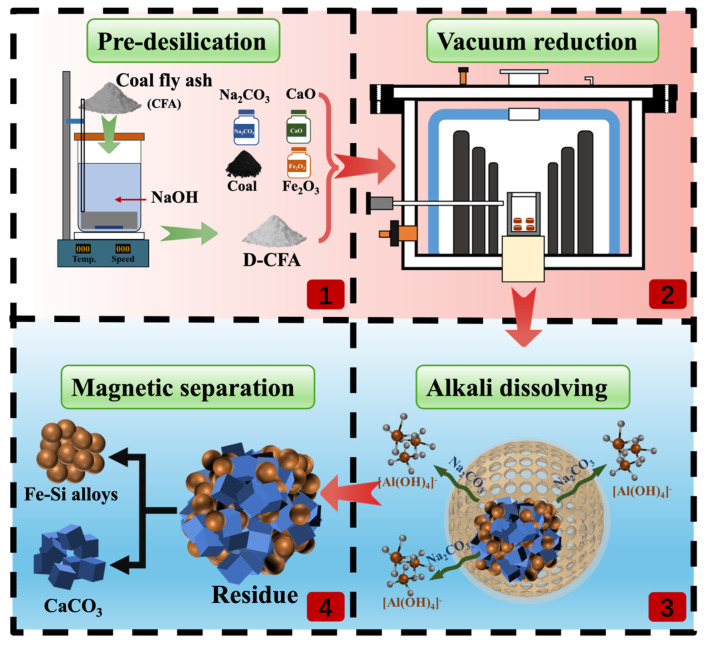
Diagram of the process. (**1**) Pre-desilication; (**2**) Vacuum carbothermic reduction; (**3**) Alkali dissolving; (**4**) Magnetic separation.

The alkali dissolving residue, comprising Fe-Si alloys and CaCO_3_, was then ground to <74 μm and subjected to wet magnetic separation using a wet magnetic separation method. The magnetic fraction (enriched in Fe-Si alloys) and the non-magnetic fraction (mainly CaCO_3_) were collected separately for further characterization. A schematic flow diagram of the whole process is shown in Figure 2.

The particle size and morphology of Fe-Si alloys in the reduced samples were examined using an optical microscope (Leica DM4P, Leica Microsystems, Wetzlar, Germany). Samples were prepared by cold mounting in epoxy resin, followed by sequential grinding with SiC papers (240, 600, 1200, and 2000 grit) and polishing with 1 μm diamond paste. Particle size analysis was performed using Image J software (version 8.1), with at least 200 particles counted for each sample.

The SEM-EDS analyses were performed using a low-vacuum field emission scanning electron microscope (FEI NOVA400 FEGSEM; FEI Company, Hillsboro, OR, USA) equipped with an energy-dispersive X-ray spectroscopy (EDS) system. Secondary electron (SE) imaging mode was employed for morphology observation. EDS analysis was used for qualitative and semi-quantitative elemental analysis, and the elemental distribution was systematically characterized.

## 3. Results and Discussion

### 3.1. Dissolution Behavior of Amorphous SiO_2_ During Pre-Desilication Process

To remove amorphous SiO_2_ and increase the Al_2_O_3_/SiO_2_ mass ratio of CFA, a pre-desilication treatment was carried out using the NaOH solution as the agent. To semi-quantitatively determine the phase composition of the raw CFA, the Reference Intensity Ratio (RIR) method was applied to the XRD data in Figure 1. The RIR method was performed using the standard (RIR = 1.0) based on the following equation:

$$W_{i}=\frac{I_{i}/RIR_{i}}{\sum_{j}(I_{j}/RIR_{j})}\times 100\%$$

where $W_{i}$ is the mass fraction of phase i, $I_{i}$ is the integrated intensity of the strongest diffraction peak of phase i, and $RIR_{i}$ is the reference intensity ratio of phase i.

The calculated results are summarized in Table 4. The raw CFA contains approximately 46.5 wt.% mullite (Al_6_Si_2_O_13_), 12.5 wt.% corundum (Al_2_O_3_), and 20.1 wt.% quartz (SiO_2_) as the crystalline phases, with the remaining 20.9 wt.% being the amorphous phase, as evidenced by the broad hump at 15–30° in the XRD pattern. The RIR method provides semi-quantitative results with an inherent error of ±10–20%.

**Figure 3:**
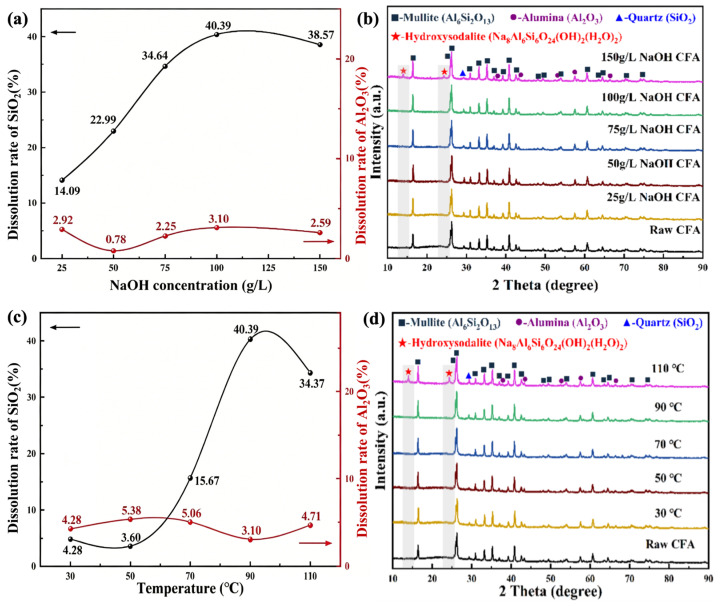
(**a**) Dissolution rate of Al_2_O_3_ and SiO_2_ under different NaOH concentration; (**b**) XRD patterns of desilicated coal fly ash under different NaOH concentrations; (**c**) Dissolution rate of Al_2_O_3_ and SiO_2_ at different dissolution temperatures; (**d**) XRD patterns of desilicated coal fly ash at different dissolution temperatures.

Figure 3a illustrates the dissolution rate of SiO_2_ in CFA as a function of NaOH concentration at 90 °C. As the NaOH concentration increased from 25 to 100 g/L, the dissolution rate of SiO_2_ rose from 14.09% to 40.39%, indicating that a portion of the SiO_2_ can be effectively removed by NaOH. This is primarily because OH^−^ ions attack the amorphous silica network, breaking Si-O-Si bonds and forming soluble sodium silicate [24]. This is supported by the XRD analysis shown in Figure 3b, where the broad diffraction peak of amorphous silica nearly disappeared after the pre-desilication process. However, when the NaOH concentration was further increased to 150 g/L, the dissolution rate of SiO_2_ dropped to 38.57%. This decline is attributed to the formation of hydroxidalite (Na_8_Al_6_Si_6_O_24_(OH)_2_(H_2_O)_2_), an insoluble compound identified in Figure 3b. The formation of this insoluble phase consumes dissolved Si and consequently reduces the efficiency. Apart from the removal of the amorphous silica, Figure 3a also shows that the dissolution rate of Al_2_O_3_ remained low (<3.5%) under all tested conditions, confirming the high selectivity of the pre-desilication step. Thus, it is demonstrated that the Al_2_O_3_ in mullite (Al_6_Si_2_O_13_) or corundum (Al_2_O_3_) is nearly insoluble in NaOH solution.

Experiments were then conducted using 100 g/L NaOH at temperatures ranging from 30 to 110 °C. As shown in Figure 3c, the dissolution rate of SiO_2_ was negligible below 50 °C (<5%), rose sharply to 40.39% at 90 °C, and then declined to 34.37% at 110 °C. The initial increase is attributed to enhanced reaction kinetics: higher temperatures increase the collision frequency between OH^−^ ions and the silica surface, reduce solution viscosity, and accelerate mass transfer. The decrease at 110 °C is again due to the formation of sodalite, as confirmed by the XRD patterns in Figure 3d, where new sodalite peaks emerge and the amorphous hump disappears. Excessive leaching at elevated temperature promotes the precipitation of hydroxysodalite, which redeposits on particle surfaces and hinders further silica removal. The dissolution rate of Al_2_O_3_ remained below 5.5% across all tested temperatures, further confirming the excellent selectivity of pre-desilication process. Based on these results, the optimal pre-desilication conditions were determined as follows: 100 g/L NaOH, 90 °C, a liquid-to-solid ratio 20, and a reaction time of 2 h. Under these conditions, the Al_2_O_3_/SiO_2_ mass ratio of CFA was successfully increased from 0.78 to 1.27, providing a significantly enriched feed for the subsequent vacuum reduction process. This result is similar to that of Bai et al. [20], in which the Al_2_O_3_/SiO_2_ ratio increased from 0.86 to 1.63 under similar conditions. It is thus proved that the pre-desilication method can effectively remove the amorphous silica in CFA.

### 3.2. Phase Transformation Mechanism of Desilicated Coal Fly Ash During Vacuum Reduction

To understand the vacuum reduction behavior of D-CFA, a thermodynamic analysis was performed using FactSage 8.1 at a system pressure of 0.001 atm. The reduction reactions of mullite (Al_6_Si_2_O_13_) in D-CFA under different additive conditions are expressed as follows:

1/4Al_6_Si_2_O_13_ + C = 3/4Al_2_O_3_ + 1/2Si + CO(g) (1)

1/10Al_6_Si_2_O_13_ + 1/5Fe_2_O_3_ + C = 3/10Al_2_O_3_ + 1/5Fe_2_Si + CO(g) (2)

1/10Al_6_Si_2_O_13_ + 1/5Fe_2_O_3_ + 3/10CaO + C = 3/10CaAl_2_O_4_ + 1/5Fe_2_Si + CO(g) (3)

**Figure 4:**
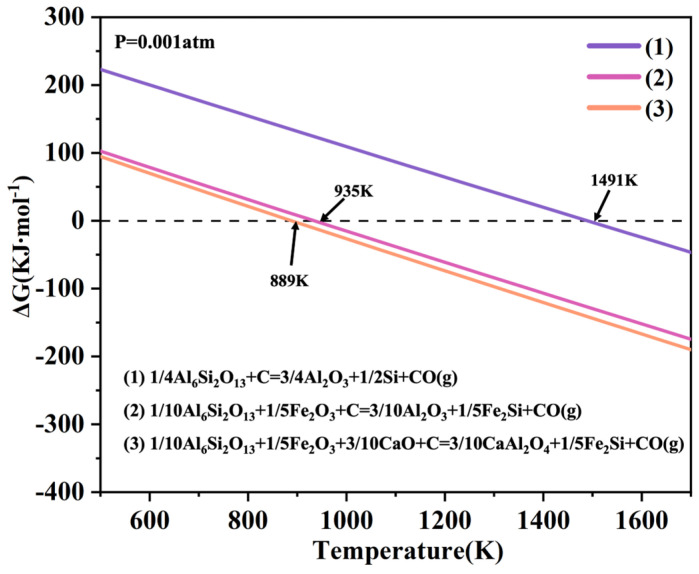
ΔG-T diagram of mullite reduction reaction at pressure of 0.001 atm.

Figure 4 presents the Gibbs free energy (ΔG)–temperature (T) diagram for the reduction reaction of mullite (Al_6_Si_2_O_13_) in D-CFA. It can be observed that, without any additives, the initial reduction temperature of mullite is 1491 K. When Fe_2_O_3_ is added, this temperature decreases to 935 K. This reduction is attributed to the formation of Fe-Si alloys by metallic iron and silicon, which lowers the activity of silicon in the reaction products. The formation of Fe-Si alloys effectively lowers the activity of silicon in the reduction products, thereby reducing the Gibbs free energy of the reduction reaction and promoting the decomposition of mullite [15,18]. Additionally, it can be also found that the initial reduction temperature of mullite further decreases to 889 K when CaO is added. The underlying reason is analogous to the previous case: CaO can combine with Al_2_O_3_ to form CaO·xAl_2_O_3_, thereby reducing the activity of Al_2_O_3_ in the final products.

**Figure 5:**
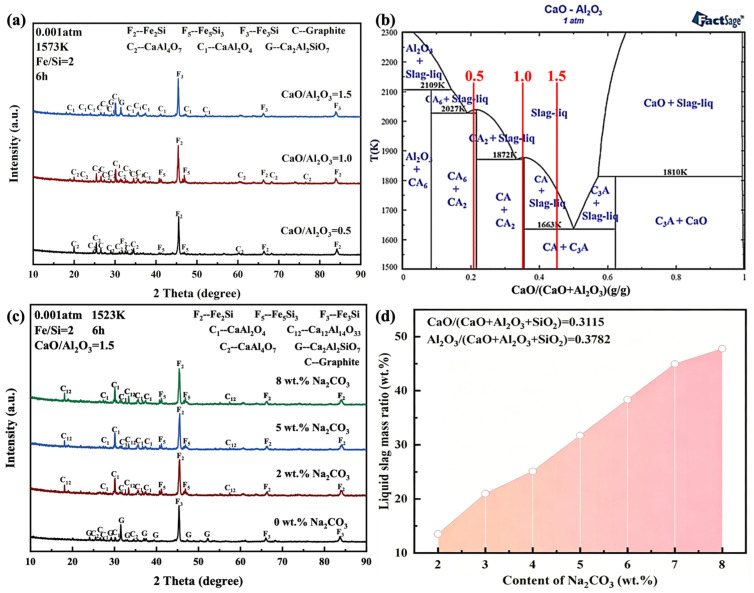
(**a**) XRD patterns of the reduced samples under different CaO/Al_2_O_3_ molar ratios; (**b**) CaO-Al_2_O_3_ binary phase diagram; (**c**) XRD patterns of reduced samples under varying sodium carbonate addition; (**d**) variation diagram of liquid phase content in the reduction system with varying sodium carbonate additions.

Figure 5a shows the XRD patterns of samples obtained from the carbothermic reduction of D-CFA with addition of Fe_2_O_3_ and CaO. The reduction was conducted at 1573 K for 6 h under a pressure of 0.001 atm. The Fe/Si molar ratio was fixed at 2, while the CaO/Al_2_O_3_ molar ratio was set to 0.5, 1.0, and 1.5, respectively. At a CaO/Al_2_O_3_ molar ratio of 0.5, the reduced sample consists of Fe_2_Si, Fe_5_Si_3_ and CaAl_4_O_7_ (CA_2_), which is largely consistent with the thermodynamic calculation results shown in Figure 4. This indicates that the mullite in D-CFA can be converted into Fe-Si alloys and CaO·xAl_2_O_3_. However, the presence of CaAl_4_O_7_ (CA_2_) differs slightly from the equilibrium phase composition. According to CaO–Al_2_O_3_ binary phase diagram, the equilibrium phase composition at a CaO/Al_2_O_3_ molar ratio of 0.5 is CaAl_4_O_7_ (CA_2_) and CaAl_12_O_19_ (CA_6_), as shown in Figure 5b. The absence of CaAl_12_O_19_ in the XRD pattern is likely due to its low content, which falls below the detection limit. When the CaO/Al_2_O_3_ molar ratio is increased to 1.0, the CaO·xAl_2_O_3_ phase consists of CaAl_2_O_4_ (CA) and CaAl_4_O_7_ (CA_2_), which agrees well with the equilibrium phase composition in Figure 5b. Further increasing the CaO/Al_2_O_3_ molar ratio to 1.5 leads to the appearance of a Ca_2_Al_2_SiO_7_ (gehlenite) phase, in addition to the Fe-Si alloys and CaAl_2_O_4_ (CA). The formation of Ca_2_Al_2_SiO_7_ (gehlenite) can be attributed to the incomplete reduction of SiO_2_ within this phase under the given conditions. This is corroborated by the composition change of Fe-Si alloys: as the CaO/Al_2_O_3_ molar ratio increases from 1.0 to 1.5, the alloy composition shifts from Fe_2_Si and Fe_5_Si_3_ to Fe_3_Si, indicating a decrease in Si concentration within Fe-Si alloys. These results confirm that the reduction of Ca_2_Al_2_SiO_7_ (gehlenite) is more difficult than that of Al_6_Si_2_O_13_ (mullite).

**Figure 6:**
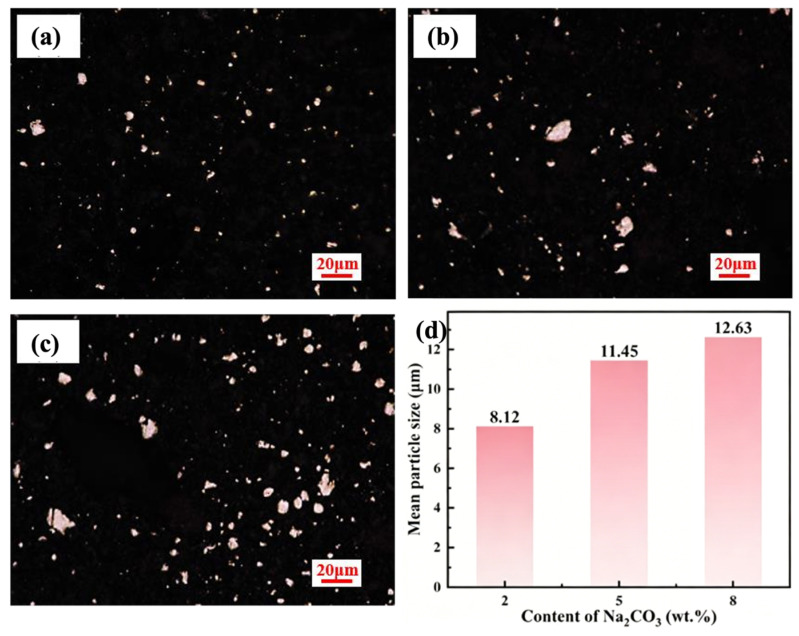
Particle size distribution of alloy particles under varying sodium carbonate additions: (**a**) 2 wt.% Na_2_CO_3_; (**b**) 5 wt.% Na_2_CO_3_; (**c**) 8 wt.% Na_2_CO_3_; (**d**) Statistical analysis of average alloy particle size.

To eliminate Ca_2_Al_2_SiO_7_ (gehlenite), Na_2_CO_3_ was introduced as an additive. In our previous study [18], the addition of Na_2_CO_3_ was shown to facilitate the decomposition of mullite in coal fly ash, proving its feasibility. Therefore, it is expected that the Na_2_CO_3_ can similarly promote the reduction of gehlenite. Figure 5c shows the XRD patterns of reduced D-CFA samples with 0, 2, 5, and 8 wt.% Na_2_CO_3_ at 1523 K for 6 h. The Fe/Si and CaO/Al_2_O_3_ molar ratios were fixed at 2 and 1.5, respectively. In the absence of Na_2_CO_3_, distinct diffraction peaks of gehlenite are observed. Upon the addition of 2 wt.% Na_2_CO_3_, these gehlenite peaks completely disappear and simultaneously, the diffraction peaks corresponding to Ca_12_Al_14_O_33_ emerge. This indicates that the SiO_2_ in Ca_2_Al_2_SiO_7_ (gehlenite) is reduced to Fe-Si alloys with the assistance of Na_2_CO_3_. The main reason for this is that low-melting-point compounds such as NaAlSi_3_O_8_ and NaAlSi_2_O_6_ are formed upon the addition of Na_2_CO_3_ [18], which generate a liquid phase and enhance mass transfer. This liquid phase accelerates the reduction kinetics, enabling the complete decomposition of gehlenite at 1523 K. Figure 5d shows the calculated liquid phase content as a function of Na_2_CO_3_ addition. At 2 wt.% Na_2_CO_3_, approximately 13.50% liquid forms; at 8 wt.%, the liquid content reaches 47.79%. The liquid phase not only facilitates the decomposition of gehlenite but also promotes the coalescence and growth of Fe-Si alloys particles. As shown in Figure 6, the average particle size of Fe-Si alloys increases from 8.12 μm to 12.63 μm when the Na_2_CO_3_ addition is raised from 2 to 8 wt.%. Large particle sizes are beneficial for the separation of Fe-Si alloys in the subsequent magnetic separation step.

### 3.3. The Alkali-Dissolving Behavior of CaO·xAl_2_O_3_ in the Reduced Sample

After vacuum reduction, the resulting product is composed primarily of CaO·xAl_2_O_3_ and Fe–Si alloys. To achieve selective extraction of alumina, the reduced sample was treated with an alkali solution, and the dissolving reaction is shown in reaction (4). During the dissolving process, CaO·xAl_2_O_3_ is dissolved by the alkali solution to form NaAl(OH)_4_, whereas the Fe-Si alloys remain undissolved [25]. Subsequently, the generated NaAl(OH)_4_ can be reacted with CO_2_ to produce Al(OH)_3_, as illustrated in reaction (5).

CaO·xAl_2_O_3_ + Na_2_CO_3_ + H_2_O → NaAl(OH)_4_ + CaCO_3_ (4)

2NaAl(OH)_4_ + CO_2_ → 2Al(OH)_3_ + Na_2_CO_3_ + H_2_O (5)

**Figure 7:**
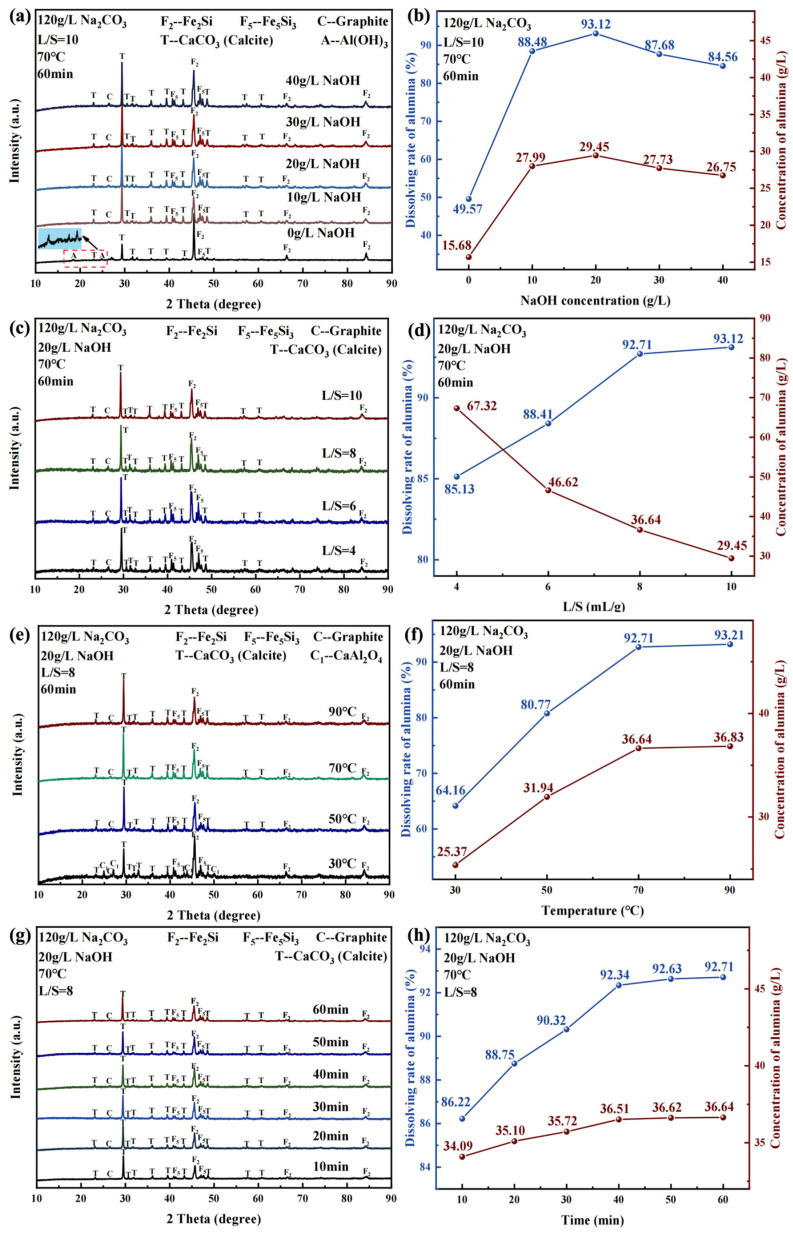
XRD patterns of dissolved residues under varying conditions: (**a**) NaOH concentrations; (**c**) liquid-to-solid ratios; (**e**) leaching temperature conditions; (**g**) leaching time conditions. Dissolving rate curves of alumina (Al_2_O_3_) under varying conditions: (**b**) NaOH concentrations; (**d**) liquid-to-solid ratios; (**f**) leaching temperature conditions; (**h**) leaching time conditions.

To clarify the dissolving mechanism of CaO·xAl_2_O_3_, a series of experiments were designed. It should be noted that the alkali-dissolving experiments were conducted using the reduced sample of D-CFA under the following conditions: reduction temperature of 1523 K, duration of 6 h, CaO/Al_2_O_3_ molar ratio of 1.5, Fe/Si molar ratio of 2, and Na_2_CO_3_ addition of 2 wt.%. Figure 7a,b illustrate the effect of NaOH concentration on the dissolving rate of alumina. As shown, when no NaOH was added to solution, the dissolving rate of alumina was only 49.57%. This is primarily attributed to the low caustic ratio (i.e., the ratio of caustic alkali to alumina in the solution), which renders the solution unstable in the absence of added NaOH. Consequently, Al(OH)_3_ precipitates unexpectedly from the solution, reducing the dissolving rate of alumina. This interpretation is supported by the XRD analysis of the dissolved residue (Figure 7b), which reveals the presence of Al(OH)_3_ when no NaOH was added. When the NaOH concentration was increased to 10 g/L, the dissolving rate of alumina rose sharply to 88.48%, and the diffraction peaks of Al(OH)_3_ disappeared. However, upon further increasing the NaOH concentration to 30 g/L, the dissolving rate of alumina decreased slightly. This decline is likely because the viscosity of the solution increases with higher NaOH concentration [26], thereby limiting mass transfer and reducing the dissolving rate. Therefore, an appropriate NaOH concentration is necessary to optimize the dissolving rate of alumina.

Figure 7c,d present the effect of liquid-to-solid (L/S) ratio on the dissolving rate of alumina. As the L/S increased from 4 to 10, the dissolving rate of alumina rose from 85.13% to 93.12%. This is because a higher L/S ratio enables more sufficient interaction between CaO·xAl_2_O_3_ and Na_2_CO_3_. However, as the L/S increased from 4 to 10, the Al_2_O_3_ concentration in the solution decreased from 67.32 g/L to 29.45 g/L, which may enhance the stability of the solution. Therefore, an L/S ratio of 8 is considered appropriate for the subsequent step (e.g., carbonation decomposition). Figure 7e,f illustrate the effect of temperature on the dissolving rate of alumina. At 30 °C, the extraction efficiency was only 64.16%, which can be attributed to the incomplete leaching of CaAl_2_O_4_, as shown in Figure 7e. When the temperature was raised to 70 °C, the dissolving rate increased to 92.71%, and the diffraction peaks of CaAl_2_O_4_ disappeared, indicating that a higher temperature facilitates the dissolving of CaAl_2_O_4_. Figure 7g,h depict the effect of time on the dissolving rate of alumina. Obviously, the dissolving rate of alumina increased rapidly during the first 40 min, reaching 92.34% at 40 min. When the dissolving time was extended from 40 min to 60 min, the dissolving rate of alumina remained nearly unchanged.

The ionic concentration of the solution after alkali dissolving is listed in Table 5. This solution was obtained under the following conditions: dissolving temperature of 70 °C, dissolving time of 60 min, liquid-to-solid (L/S) ratio of 8, Na_2_CO_3_ concentration of 120 g/L, and NaOH concentration of 20 g/L. As shown in the table, the concentrations of Al^3+^ and Si^4+^ are 19.40 g/L and 0.01348 g/L, respectively, corresponding to a siliceous modulus of 1270. Furthermore, the concentrations of Fe^3+^ and Ca^2+^ are 0.08031 g/L and 0.00179 g/L, respectively, indicating that the solution has low impurity levels and is suitable for alumina extraction.

The alumina dissolution rate of 92.71% achieved in this work is similar to that of Yu et al. [15] and Rao et al. [17], where the alumina dissolution rates are 93.8% and 94.62%, respectively. However, the alkali dissolving in Yu et al. [15] and Rao et al. [17] was carried out in an autoclave, while the dissolving condition in this work is atmospheric. This indicates that the energy consumption for alkali dissolving can be reduced in this work.

### 3.4. Magnetic Separation of Fe-Si Alloys in the Dissolved Residue

**Figure 8:**
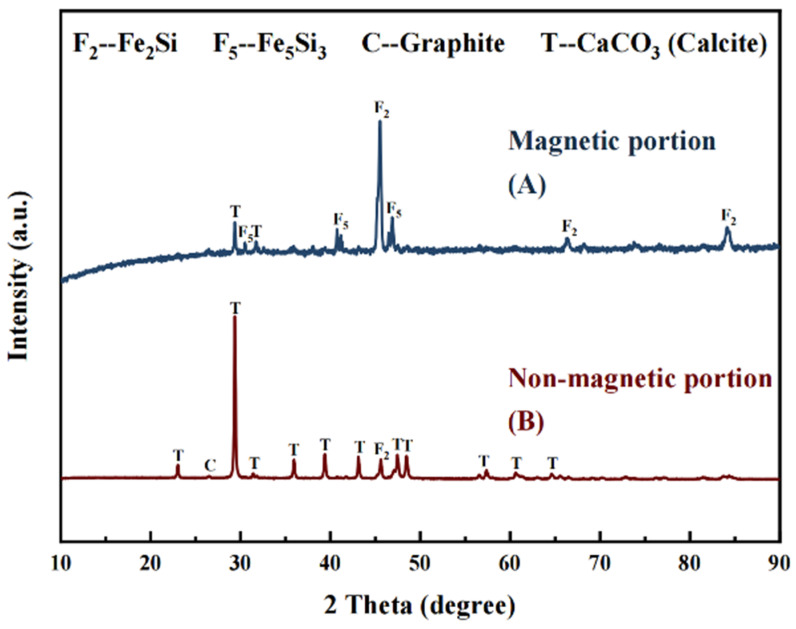
XRD patterns of magnetic and non-magnetic samples obtained by magnetic separation of dissolved residue.

**Figure 9:**
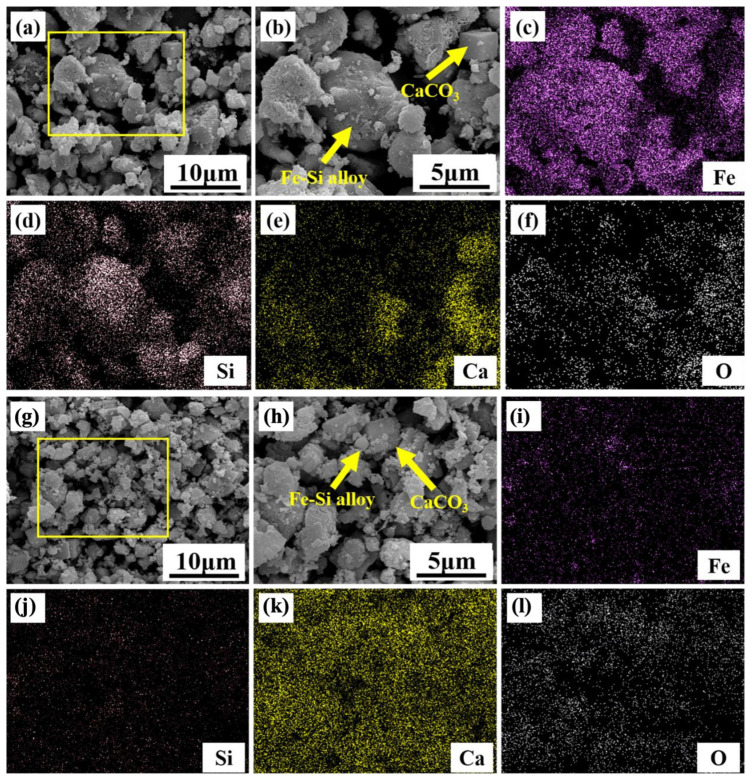
(**a**–**f**) SEM images and EDS analysis of the magnetic samples; (**g**–**l**) SEM images and EDS analysis of the non-magnetic samples.

After alkali dissolving, the residue consists primarily of Fe-Si alloys and CaCO_3_. Given the ferromagnetic nature of the Fe-Si alloys, magnetic separation was employed to recover the Fe-Si alloys from the residue. Prior to magnetic separation, the residue was ground to a particle size of <74 μm. Figure 8 presents the XRD patterns of the magnetic and non-magnetic fractions. Evidently, the magnetic fraction is predominantly composed of Fe-Si alloys, whereas the non-magnetic fraction is mainly CaCO_3_ (calcite), indicating that magnetic separation can effectively recover Fe-Si alloys. Quantitative chemical analysis results for the two fractions are summarized in Table 6. The magnetic product contains 65.10 wt.% Fe and 16.58 wt.% Si, while the non-magnetic product contains 84.61 wt.% CaCO_3_. Approximately 12.54 wt.% CaCO_3_ reports to the magnetic fraction, and 5.96 wt.% Fe enters the non-magnetic portion, suggesting that the Fe-Si alloys and CaCO_3_ cannot be separated completely. Figure 9 shows the SEM-EDS analysis results of the magnetic and non-magnetic fractions. It can be seen that some CaCO_3_ particles are observed in the magnetic portion, likely due to their adherence to the surface of Fe-Si alloys particles. This phenomenon of fine particle adherence is consistent with the observation of iron oxide/hydroxide adherence to calcite surfaces reported in the literature [27]. Additionally, tiny Fe-Si alloys particles (<1 μm) are present in the non-magnetic portion. This is attributed to their weak magnetizing force, which prevents them from being captured during the magnetic separation. Therefore, an optimized magnetic separation process is necessary for the recovery of Fe-Si alloys from the residue in future work.

### 3.5. Material and Energy Consumption Analysis

To evaluate the material and energy consumption of the process with and without pre-desilication step, a preliminary calculation was conducted based on the optimal experimental conditions (pre-desilication: 100 g/L NaOH, 90 °C, 2 h; vacuum reduction: 1523 K, Fe/Si molar ratio = 2, CaO/Al_2_O_3_ molar ratio = 1.5, 2 wt.% Na_2_CO_3_, 6 h). The calculation was performed based on material and energy balances [28], assuming an alumina recovery of 90% in the alkali dissolving step for both CFA and D-CFA feeds, targeting the extraction of 1000 kg alumina.

Table 7 shows the total material input and energy consumption required for the vacuum reduction stage when using CFA and D-CFA as the raw materials, respectively. It is evident that pre-desilication significantly reduces the required amount of Fe_2_O_3_ additive from 3731 kg to 2278 kg, owing to the removal of a large fraction of amorphous silica. Consequently, the total material input decreases from 9742 kg to 6795 kg, achieving a reduction of 30.25%. The energy consumption for the vacuum reduction process, calculated at a comprehensive thermal efficiency of 70%, drops from 5.97 × 10^10^ J to 3.87 × 10^10^ J, corresponding to a reduction of 35.18%. These improvements are primarily attributed to the lower silica content in D-CFA, which reduces the demand for iron oxide to form a separable Fe-Si alloys phase. The substantial savings in material and energy not only lower production costs but also enhance the overall environmental and economic feasibility of the integrated process.

## 4. Conclusions

In this study, a novel process integrating pre-desilication, vacuum reduction, and alkali dissolving was proposed. The dissolution behavior of amorphous SiO_2_ during the pre-desilication process was investigated. Additionally, the phase transformation mechanism of desilicated coal fly ash during vacuum reduction was examined. Furthermore, the dissolving behavior of CaO·xAl_2_O_3_ and the magnetic separation of Fe-Si alloys were discussed. Based on this analysis, the material and energy consumption with and without the pre-desilication step were calculated and compared. The key conclusions are as follows:

(1) During the pre-desilication process, the amorphous silica in coal fly ash can be removed effectively by NaOH solution. Under optimal conditions, e.g., 100 g/L NaOH, 90 °C, 2 h, the dissolution rate of silica achieved 40.39%, whereas the dissolution rate of alumina was only 3.10%, increasing the Al_2_O_3_/SiO_2_ mass ratio from 0.78 to 1.27. This pre-treatment step enriched the alumina content and laid the foundation for efficient subsequent processing.
(2) Thermodynamic and experimental studies confirmed that the stable mullite (Al_6_Si_2_O_13_) in desilicated coal fly ash (D-CFA) can be converted into the Fe-Si alloys and CaO·xAl_2_O_3_ with the assistance of Fe_2_O_3_ and CaO during the vacuum carbothermic reduction. The gehlenite (Ca_2_Al_2_SiO_7_) that appeared in the reduced sample can be eliminated completely by adding some Na_2_CO_3_. The resulting CaO·xAl_2_O_3_ can subsequently be processed to extract the alumina, and an alumina dissolving rate of over 90% was achieved.
(3) The total material input and energy consumption required for the vacuum reduction stage when using CFA and D-CFA as the raw materials were calculated based on the material and energy balances. It is evident that the reduction in material and energy consumption for the vacuum reduction step is 30.25% and 35.18%, respectively, when D-CFA is used as the raw material.

## Figures and Tables

**Table 1:** Chemical composition of coal fly ash (wt.%).

Composition	Al_2_O_3_	SiO_2_	CaO	Fe_2_O_3_	TiO_2_	Na_2_O
Content	38.97	49.89	5.01	1.92	1.57	0.13

**Table 2:** Component analyses of the reducing agents used in the experiment.

Sample	Proximate Analysis (wt.%)				Ultimate Analysis (%)				
Coal	Fixed carbon	Ash	Volatile matter	Moisture	C_ad_	H_ad_	O_ad_	N_ad_	S_t,ad_
	71.22	7.87	19.29	1.62	81.11	4.93	2.55	1.50	0.42

**Table 3:** PDF/JCPDS card numbers used for phase identification.

Phase	PDF/JCPDS Card No.
Mullite (Al_6_Si_2_O_13_)	PDF#15-0776
Quartz (SiO_2_)	PDF#46-1045
Corundum (Al_2_O_3_)	PDF#10-0173
CaAl_4_O_7_	PDF#70-0134
CaAl_2_O_4_	PDF#70-0135
Ca_12_Al_14_O_33_	PDF#48-1882
Fe_2_Si	PDF#35-0502
Fe_5_Si_3_	PDF#45-1207
Fe_3_Si	PDF#65-3497
Ca_2_Al_2_SiO_7_	PDF#35-0755
CaCO_3_ (Calcite)	PDF#05-0586
NaAlSiO_4_	PDF#35-0424
Al(OH)_3_	PDF#10-0173
AlOOH	PDF#21-1307
SiC	PDF#29-1129

**Table 4:** Relative proportions of crystalline phases in raw CFA (calculated by RIR method).

Mineral Phase	Chemical Formula	Content (wt.%)
Mullite	Al_6_Si_2_O_13_	46.5
Corundum	Al_2_O_3_	12.5
Quartz	SiO_2_	20.1
Amorphous (glassy) phase	—	20.9

**Table 5:** Ionic concentration of the solution from the dissolving process.

Element	Al^3+^	Na^+^	Fe^3+^	Si^4+^	Ca^2+^
Concentration (g/L)	19.40	52.69	0.08031	0.01348	0.00179

**Table 6:** Chemical composition of magnetic and non-magnetic samples (wt.%).

Composition	Fe	Si	CaCO_3_	Al_2_O_3_
Magnetic Sample	65.10	16.58	12.54	2.57
Non-magnetic Sample	5.96	2.66	84.61	2.43

**Table 7:** Statistics of energy and material consumption for the vacuum reduction process.

Item	Raw CFA	Pre-Desilicated
Raw material/kg	2851	2263
Fe_2_O_3_ added/kg	3731	2278
Bituminous coal/kg	2197	1352
CaO added/kg	772	769
Na_2_CO_3_ added/kg	191	133
Total material input/kg	9742	6795
Energy consumption/J	5.97 × 10^10^	3.87 × 10^10^

## Data Availability

The original contributions presented in this study are included in the article. Further inquiries can be directed to the corresponding author.

## Abbreviations

The following abbreviations are used in this manuscript:

CFA	Coal fly ash
D-CFA	Desilicated coal fly ash
XRD	X-ray diffraction
XRF	X-ray fluorescence
ICP-OES	Inductively coupled plasma–optical emission spectrometry
SEM-EDS	Scanning electron microscopy with energy-dispersive spectroscopy
PTFE	Polytetrafluoroethylene
ad	Air-dried basis
t,ad	Total sulfur on air-dried basis
